# Non-invasive in vivo imaging of cardiac stem/progenitor cell biodistribution and retention after intracoronary and intramyocardial delivery in a swine model of chronic ischemia reperfusion injury

**DOI:** 10.1186/s12967-017-1157-0

**Published:** 2017-03-13

**Authors:** María Collantes, Beatriz Pelacho, María José García-Velloso, Juán José Gavira, Gloria Abizanda, Itziar Palacios, Luis Rodriguez-Borlado, Virginia Álvarez, Elena Prieto, Margarita Ecay, Eduardo Larequi, Iván Peñuelas, Felipe Prósper

**Affiliations:** 1 Department of Nuclear Medicine, IdisNA, Clínica Universidad de Navarra, Avda. Pío XII, 31080 Pamplona, Spain; 2 Center for Applied Medical Research (CIMA) Cell Therapy Area, IdiSNA, Universidad de Navarra, Avda. Pío XII, 31080 Pamplona, Spain; 3 Department of Cardiology and Cardiovascular Surgery, IdiSNA, Clínica Universidad de Navarra, Avda. Pío XII, 31080 Pamplona, Spain; 4 Coretherapix, Santiago Grisolía, n° 2 Parque Científico de Madrid, Tres Cantos, 28760 Madrid, Spain; 5 Small Animal Imaging Research Unit, Center for Applied Medical Research (CIMA), Universidad de Navarra, Pamplona, Spain; 6 Hematology and Cell Therapy, IdiSNA, Clínica Universidad de Navarra, Avda. Pío XII, 31080 Pamplona, Spain

**Keywords:** Myocardial infarction, Cardiac stem/progenitor cells (CSC), PET/CT, Cell tracking, Preclinical pig model

## Abstract

**Background:**

The safety and efficacy of cardiac stem/progenitor cells (CSC) have been demonstrated in previous preclinical and clinical assays for heart failure. However, their optimal delivery route to the ischemic heart has not yet been assessed. This study was designed to determine by a non-invasive imaging technique (PET/CT) the biodistribution and acute retention of allogeneic pig CSC implanted by two different delivery routes, intracoronary (IC) and intramyocardial (IM), in a swine preclinical model of chronic ischemia–reperfusion.

**Methods:**

Ischemia–reperfusion was induced in six Goettingen hybrid minipigs by 90 min coronary artery occlusion followed by reperfusion. Thirty days later, animals were allocated to receive IC (n = 3) or NOGA^®^-guided IM injection (n = 3) of 50 million of ^18^F-FDG/GFP-labeled allogeneic pig CSC. Acute retention was quantified by PET/CT 4 h after injection and cell engraftment assessed by immunohistochemical quantification of GFP^+^ cells three days post-injection.

**Results:**

Biodistribution of ^18^F-FDG-labeled CSC was clearly visualized by PET/CT imaging and quantified. No statistical differences in acute cell retention (percentage of injected dose, %ID) were found in the heart when cells were administered by NOGA^®^-guided IM (13.4 ± 3.4%ID) or IC injections (17.4 ± 4.1%ID). Interestingly, engrafted CSC were histologically detected only after IM injection.

**Conclusion:**

PET/CT imaging of ^18^F-FDG-labeled CSC allows quantifying biodistribution and acute retention of implanted cells in a clinically relevant pig model of chronic myocardial infarction. Similar levels of acute retention are achieved when cells are IM or IC administered. However, acute cell retention does not correlate with cell engraftment, which is improved by IM injection.

**Electronic supplementary material:**

The online version of this article (doi:10.1186/s12967-017-1157-0) contains supplementary material, which is available to authorized users.

## Background

Cardiovascular diseases are the main cause of morbidity and mortality worldwide, leading to approximately 17 million deaths per year. Unfortunately, predictions are not favorable and it is estimated that by 2030 about 24 million people will die from cardiovascular diseases, representing 42% of deaths world-wide [1]. Alternative therapies based on the application of stem cells have been examined in preclinical models and clinical trials in patients with heart failure [2, 3]. Among the different cell types employed, cardiac stem/progenitor cells (CSC) have demonstrated a remarkable potential to induce a beneficial effect after implantation in rodent and pig models of myocardial infarction (MI) [4–6]. Furthermore, their therapeutic effect has been shown in a clinical trial performed in patients with heart failure [7].

Despite the reported benefits of stem cells, some of the divergent results observed in clinical trials have been explained by the low level of cell engraftment and the lack of precise knowledge of the mechanisms of cell mediated cardiac repair [8]. Delivery of stem cells either by an intracoronary (IC) route or directly by intramyocardial injection (IM) has been associated with different degrees of cell retention (reviewed in [3]). In this sense, the use of three-dimensional electromechanical mapping (NOGA^®^) as a delivery system that allows targeting to the viable myocardium could favor cell engraftment [9, 10] and could be of great use specially in the case of those patients where the coronary artery might be blocked impeding an IC delivery.

In order to compare different delivery approaches and cell types, methods are needed for monitoring stem cell grafts non-invasively, with sufficiently high sensitivity and specificity to determine the fate of transplanted cells. Molecular imaging techniques for in vivo cell tracking, such as labeling of cells with 2-deoxy-2-[^18^F]fluoro-d-glucose (^18^F-FDG) that can be subsequently followed by means of positron emission tomography (PET), have been explored [11, 12]. This method allows cell tracking for a sufficient period of time (hours) to assess acute cell retention. Moreover, PET permits quantification of the images where an estimation of cell numbers in a specific organ can be obtained. This technique has successfully been applied for cell tracking in small and large animal models of MI [13–18].

In this study, the acute retention of allogeneic pig CSC has been assessed in a swine model of chronic MI using a cutting-edge PET/CT hybrid scanner. Acute retention of CSC was comparatively quantified when using two different cell delivery routes (IC and NOGA^®^-guided IM) and correlated with CSC engraftment three days post-implantation by immunohistological detection of CSC.

## Methods

### Pig cardiac stem/progenitor cells isolation, GFP labeling and phenotypic characterization

CSC isolation, culture, characterization and green fluorescent protein (GFP) lentiviral transduction were performed by Coretherapix SLU as previously reported [6]. Briefly, starting material (~1 g of cardiac tissue) obtained from healthy female Gottingen minipig hearts was digested by combining mechanical dissociation with enzymatic digestion [collagenase type 2 (Worthington Biochemical Corporation)] to obtain a cell suspension. Cell suspension was immunodepleted of CD45-positive cells and immunoselected for CD117 (c-kit) using magnetic microbeads coupled with specific antibodies (Miltenyi Biotech). The resulting cells were seeded on pig gelatin (Sigma-Aldrich) coated plates and cultured in low O_2_ conditions (3%) using isolation medium (DMEM/F12 with 10% FBS, l-glutamine, penicilline–streptomycine, insulin-transferrin-selenium (ITS) and bFGF, IGF-II, EGF and hEPO recombinant growth factors (Invitrogen™, Peprotech and Sigma-Aldrich)). One week after seeding, the medium was replaced by growing medium (DMEM/F12 and Neurobasal medium (1:1) supplemented with 10% FBS, l-Glutamine, Pen-Strep, ITS, growth factors (bFGF, IGF-II, EGF) together with B27, N2 and β-mercaptoethanol (Invitrogen™). Cells were expanded over 3 passages and then cryopreserved in medium with 5% of DMSO.

Cells used in this assay were Green Fluorescent Protein (GFP) labeled at passage 4 with a bicistronic, self-inactivating, lentiviral vector (pSIN-EF1-α-GFP-IRES-Puro) that expresses GFP and puromycin resistance genes under the control of an EF1α eukaryotic promoter. Lentiviral vector transduction of CSC, at passage P3/1, was performed by spinoculation (centrifugation at 1000×*g* for 1 h at 34 °C) of 1.7 × 10^6^ cells with 4.3 ml of lentiviral supernatant supplemented with 8 µg/ml of polybrene. Multiplicity of infection (MOI) was estimated to be 2.5 TU/cell. Transduction efficiency was measured by quantification of the GFP expression in positive cells compared to non-transduced CSC. GFP expression was analyzed in an EPICS^®^ XL™ (Beckman Coulter) flow cytometer. GFP brightness, acceptable for in vivo detection, was also visually evaluated by fluorescence microscopy (Nikon Eclipse TS100). Finally, phenotypic analysis of surface markers on GFP-labeled CSC was performed by resuspending 2 × 10^5^ cells in 100 µl of ice cold PBS containing 1% BSA and 1% human serum to be stained for 40 min at 4 °C in the dark and orbital shaker with combinations of following purified or conjugated mAb: purified CD11R3; purified CD29 and SLA-II (VMRD, Pullman, WA, USA) and PE-conjugated CD45, FITC-conjugated CD90 and CD105 (BD Biosciences, San Jose, CA, USA). Background fluorescence was assessed using appropriate isotype- and fluorochrome-matched control mAbs (BD Biosciences) in parallel. Afterwards the cells were washed twice with PBS 0.1%-BSA buffer. Secondary antibody PE-conjugated anti mIgG1/mIgG2b (BD Biosciences) were added when needed for 15 min at 4 °C, dark environment and shaking, followed by 2 cycles of cell washing. Finally, cells were resuspended in PBS 0.1% BSA buffer to be analyzed by flow cytometry (Epics XL-MCL flow cytometer, Beckman Coulter, Fullerton, CA, USA) and FCS Express software.

### ^18^F-FDG labeling of pig cardiac stem/progenitor cells

^18^F-FDG was optimized for labeling of 50 × 10^6^ cells, which were suspended in glucose-free DMEM supplemented with 5% human serum albumin and incubated with ^18^F-FDG (370 MBq/ml) at room temperature for 60 min. Cells were then washed twice with PBS and resuspended in DMEM for implantation. Supernatant and pellet (cells) radioactivity were measured in a dose calibrator. A trypan blue viability test was performed to calculate cell viability before and after radiolabeling. To assess ^18^F-FDG efflux from CSC, the variation in radioactivity in the supernatant was measured at 60, 90 and 120 min post-labeling. This experiment was repeated four times.

### MI and cell administration in adult Gottingen minipigs

Adult Goettingen hybrid minipigs (60–80 kg, n = 6) were procured from our breeding center (GLP accredited center at the University of Navarra, Spain) according to the legal and ethical requirements of EU legislation. In each procedure, swine were pre-medicated, induced, intubated and mechanically ventilated. Postoperatively, all animals received opioid patches, NSAIDs and antibiotics.

MI (ischemia–reperfusion) was provoked as previously described by our group [19, 20]. Briefly, an introducer sheath was placed by dissection in the left carotid artery and adjunct agents were intravenously administered prior to introducing the catheter. Under fluoroscopic guidance, a 7fr guiding catheter was positioned in the left coronary ostium and MI was induced by selectively delivering a balloon angioplasty catheter (via a microcatheter advanced through the guiding catheter to the anterior descendent artery (ADA) that was inflated for 90 min. Coronary occlusion was demonstrated by coronary angiography and ST-segment changes in the electrocardiogram. Adjunct agents and advanced life support were used when needed. Finally, the delivery catheter was removed, the carotid artery ligated, and the cut down site sutured.

Thirty days post-MI, 50 million of allogeneic pig CSC-GFP^+^ previously labeled with ^18^F-FDG (1.45 ± 0.8 MBq/kg of ^18^F-FDG labelled cells) were transplanted by two different methods: percutaneously or IC. Percutaneous transplantation (n = 3) was performed by a NOGA injection catheter, advanced from the femoral artery and across the aortic valve. NOGA mapping guided intra-myocardial transplantation was performed using the Myostar injection catheter (Bioscience-Webster) based on the generated 3D map of the heart; 50 million cells were resuspended in 9 ml of DMEM culture media and a total of 30 injections (300 μl/per injection) were slowly applied at each transplantation site. For intracoronary injection, cells were resuspended in 20 ml of DMEM.

### PET/CT acquisition and analysis

Animals were imaged on a Biograph mCT PET scanner (Siemens Medical Solutions, Hoffman Estates, IL, USA) 4 h after cell administration. A detailed description of this PET/CT system and its performance characterization can be found elsewhere [21]. This scanner allows point spread function depth-dependent resolution recovery and time-of-flight acquisition and reconstruction [22]. The acquisition protocol consisted of a whole-body CT scan for attenuation correction and a 3D whole-body PET emission scan (5–6 bed positions, 10 min per bed position). Images were reconstructed with the 3D-OP-OSEM + PSF algorithm with TOF with 3 iterations and 21 subsets respectively (matrix sizes 200 × 200 and a Gaussian post- reconstruction filter with FWHM = 2 mm).

Visual analysis of the CSC biodistribution was performed using a workstation with True D software (Siemens Healthcare, Erlangen, Germany). For the quantitative analysis, all studies were exported and analyzed using the PMOD software (PMOD Technologies Ltd., Adliswil, Switzerland). A total-body volume of interest (VOI) was drawn to measure the total activity in the body, expressed in KBq. Additional VOIs were drawn over heart, lungs, liver, spleen, kidneys and bladder. All the VOIs were created over CT images and then copied and pasted onto the corresponding PET images. The percentage of injected dose in the organs (%ID) was then calculated as the ratio of activity in the organ VOI and activity in the total-body VOI. Afterwards, considering the physiological distribution of ^18^F-FDG [23], the percentage of cell retention in the heart was calculated assuming that the signal produced by the cells was located exclusively at the heart and the lungs.

### Histological processing and immunostaining

Three days after CSC administration, animals were sacrificed with pentobarbital and a saturated solution of potassium chloride, and the heart was excised. Explanted tissues were fixed in formalin, cut in 2–4 cm^2^ blocks and paraffin embedded for histological analysis. Location of MI was visually assessed. Sampling of tissues consisted of scar surrounded by a ring of viable myocardium. After paraffin embedding, 5 μm sections were prepared from all blocks and stained for GFP to quantify the presence of engrafted CSC in the infarct and peri-infarct areas, as previously described [19]. Briefly, cell detection was performed by immunohistochemical methods. A rabbit anti-GFP (Invitrogen) was diluted 1:500 in TBS and used as first antibody. EnVision™-HRP conjugated system (Dako) was used as secondary reagent. High-power field images were acquired and GFP positive cells quantified using Image J software. A minimum of 40 sections per animal were analyzed for analysis. All microphotographs were obtained on a Nikon Eclipse E800 microscope and analyzed with a computarized system (Axiovision 4.6, Zeiss, Germany).

### Statistical analysis

All data are expressed as mean ± SD. Comparisons between IC and IM data were performed using the Mann–Whitney U test. Statistical analysis was performed with the StataIC 12 software and differences were considered statistically significant when p < 0.05.

## Results

### Pig cardiac stem/progenitor cells isolation and characterization

**Fig. 1:**
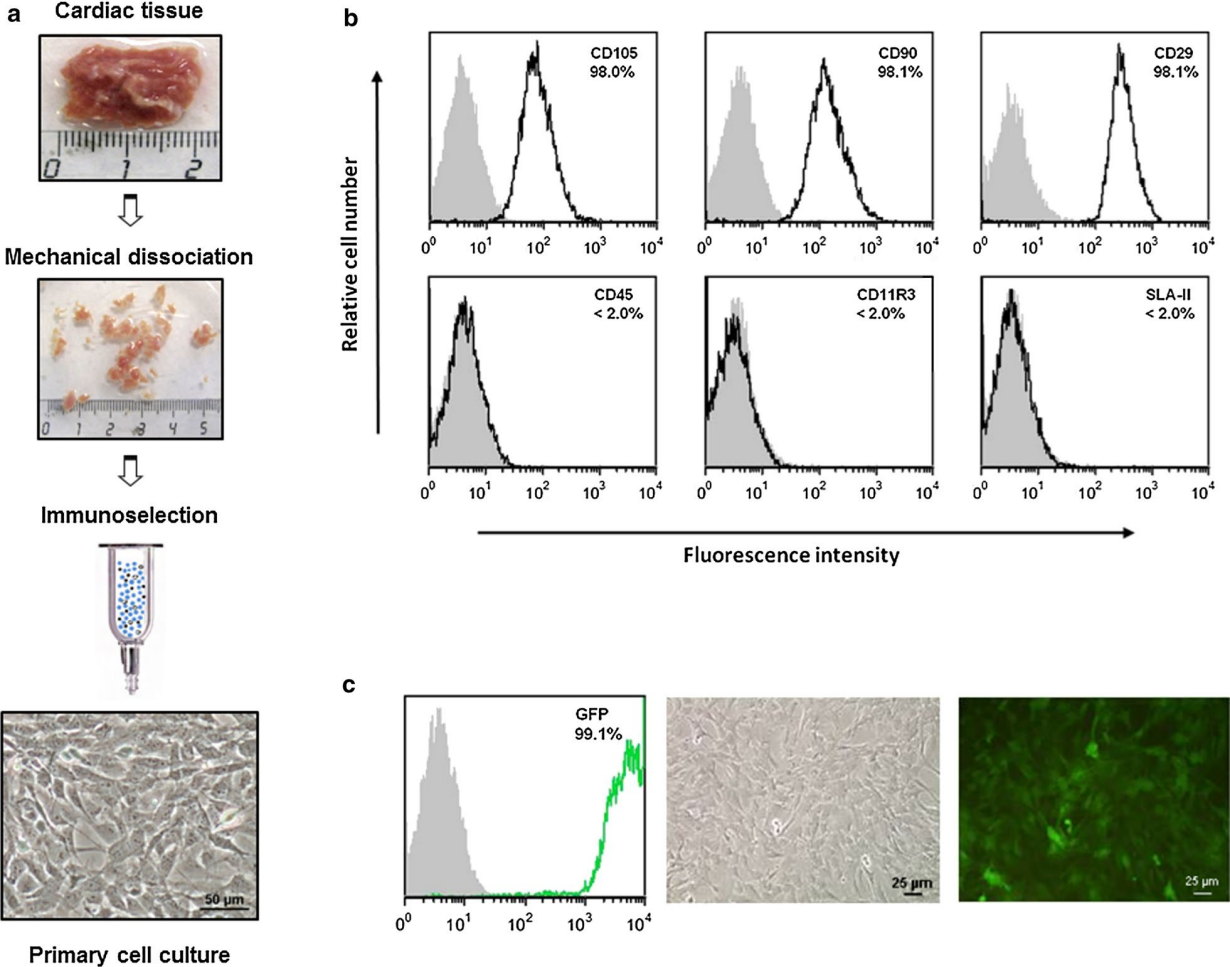
Pig CSC isolation and characterization: Scheme of the process followed to isolate pig CSC from cardiac tissue (**a**). Phenotypic characterization of CSC by flow cytometry. The percentage of positive cells with respect to the matched isotype control is indicated (**b**). Lentiviral GFP expression in CSC administered into infarcted pigs quantified by flow cytometry was 99.00 ± 0.04%. Representative pictures of CSC culture in dark field and under fluorescence microscopy (**c**)

Pig CSC were isolated from the cardiac tissue of Gottingen pigs and maintained in culture as previously reported [6]. CSC had an average size below 14 µm. Phenotypic characterization by flow cytometry revealed expression of CD105, CD90 and CD29 markers and no expression of the hematopoietic lineage markers CD45, CD11R3 or SLA-II (Fig. 1).

### Radiolabeling of CSCs with ^18^F-FDG

Radiolabeling did not induce a significant change in cell viability (−2 ± 4.6% of variation between CSC incubated with ^18^F-FDG and control cells). Efflux of ^18^F-FDG from cells was 32.4 ± 3.4% at 60 min. This release remained stable at 90 min (29.3 ± 2.2%) and 120 min (31.2 ± 2.5%).

### Cell biodistribution and acute retention after IM and IC injection

**Fig. 2:**
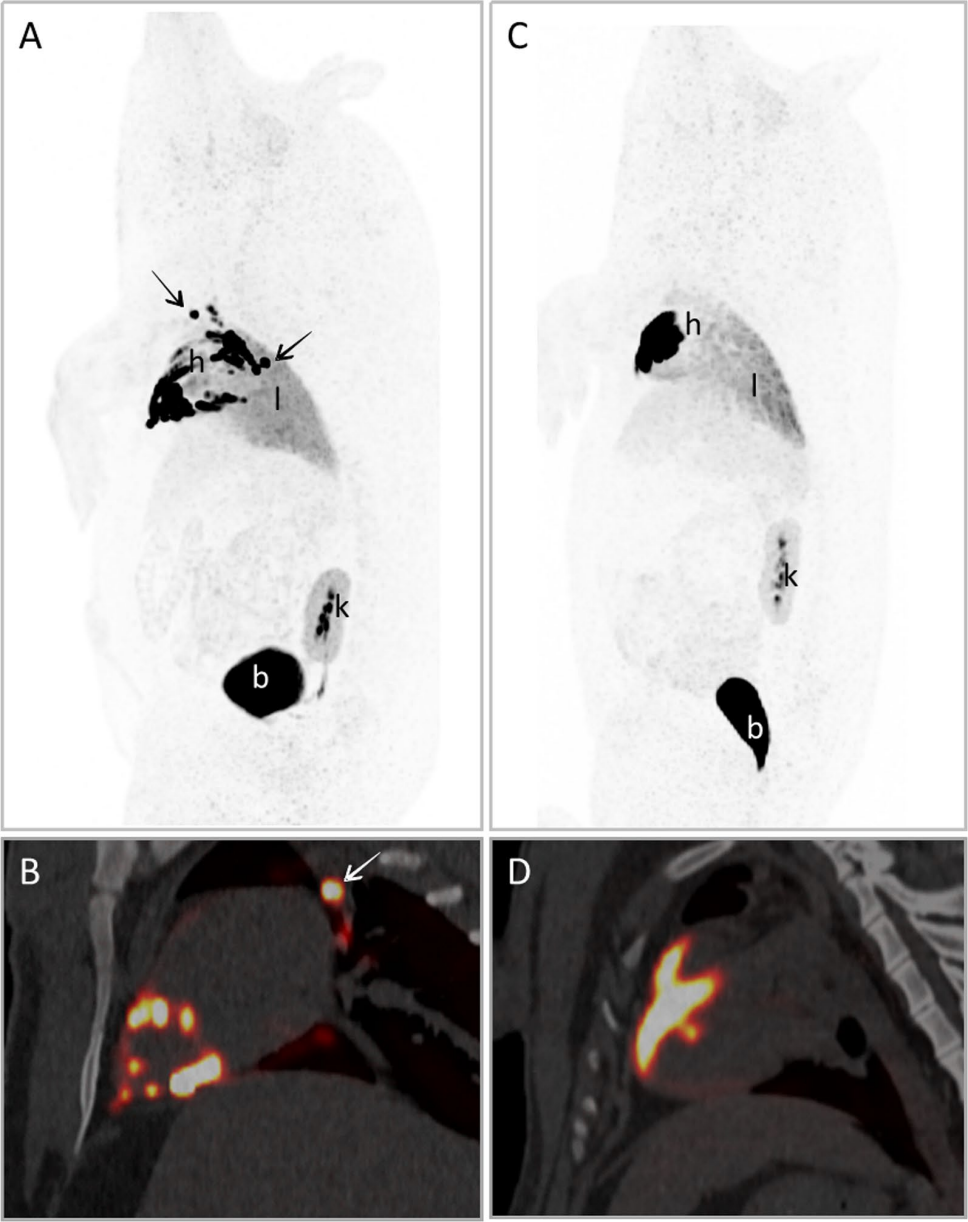
PET/CT images after ^18^F-FDG labeled CSC administration: Images of IM (**A**, **B**) and IC (**C**, **D**) administration of ^18^F-FDG-labeled CSC in pigs. **A** and **C** correspond to PET maximal intensity projection (MIP) images, showing the distribution of ^18^F-FDG activity over the entire body of the animals. **B** and **D** are sagittal sections of PET/CT images only in the heart area. In IM images, a spot-pattern uptake can be clearly observed over myocardial wall (*h*), whereas IC administration showed a diffuse uptake. ^18^F-FDG activity could also be clearly detected in bladder (*b*), kidneys (*k*) and lungs (*l*). *Arrows* point to lymph nodes with high ^18^F-FDG uptake

**Fig. 3:**
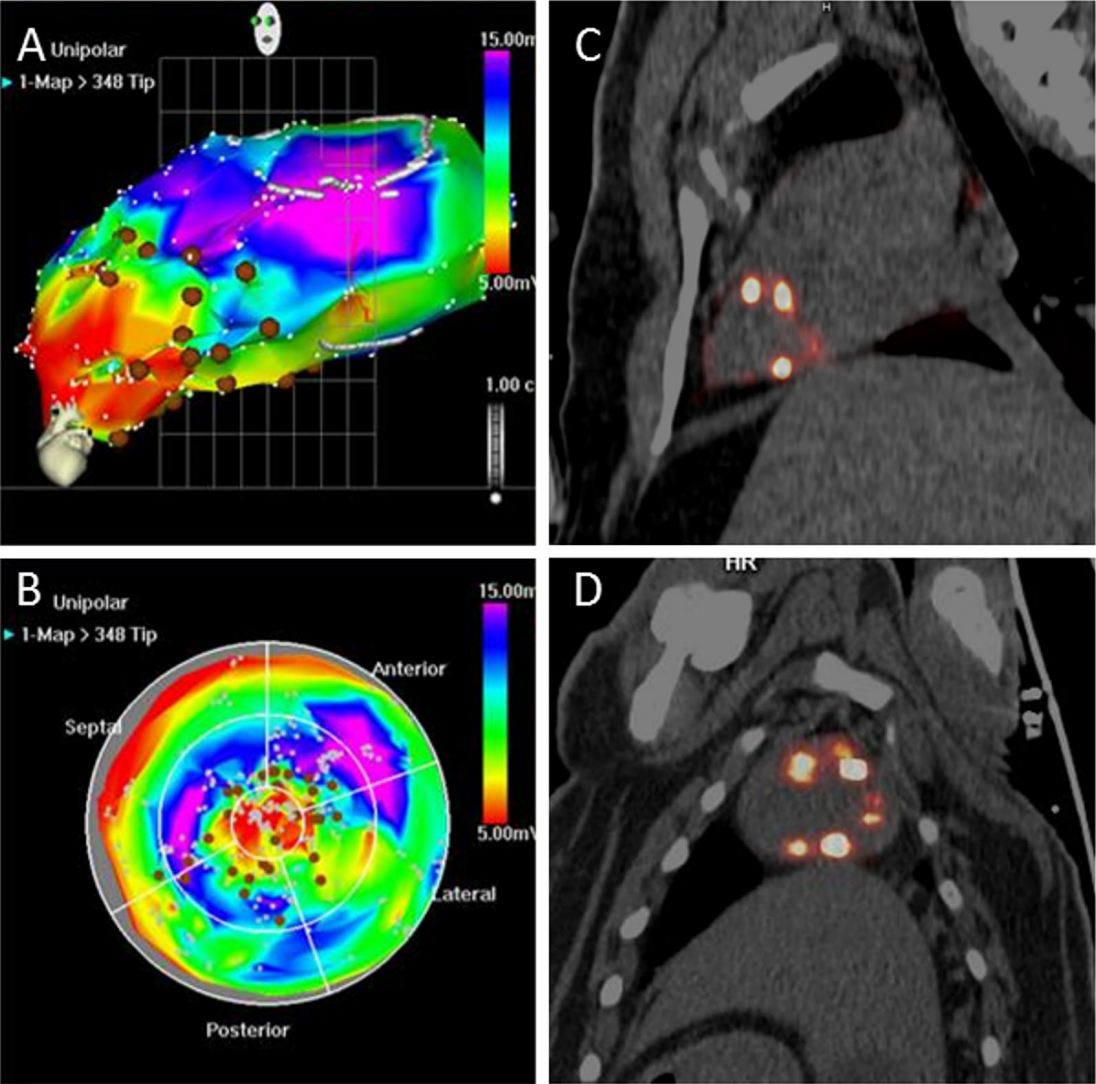
Correspondence between NOGA^®^ and PET/CT images in IM administration: Three-dimensional views derived by NOGA^®^ endocardial mapping showing peri-infarct cell injections (**A**, **B**, *brown spots*). PET/CT images correspond to sections (not 3D projections) in the vertical long (**C**) and short axis (**D**) of the same pig. Focal uptake spots showed in ^18^F-FDG PET/CT images correspond to the cellular injections

Visual analysis of PET images showed the biodistribution of radioactivity 4 h after CSC administration, demonstrating a high ^18^F-FDG activity in the heart (Fig. 2; see also Additional file 1: Video 1, Additional file 2: Video 2). The distribution of cells after IC administration showed a diffuse pattern through the vascular territory whereas IM delivery via NOGA^®^ led to a focal uptake pattern over the myocardial wall, corresponding to the cell injection sites (Fig. 3). Radioactivity was also clearly observed in the lungs, whereas liver and spleen showed a slight signal. Due to the efflux of ^18^F-FDG from cells, physiological excretion of free ^18^F-FDG was detected in kidneys, mainly in renal medulla, and bladder. Additionally, in four out of six animals, a high radioactivity concentration was detected in some mediastinal lymph nodes (Fig. 2).

**Fig. 4:**
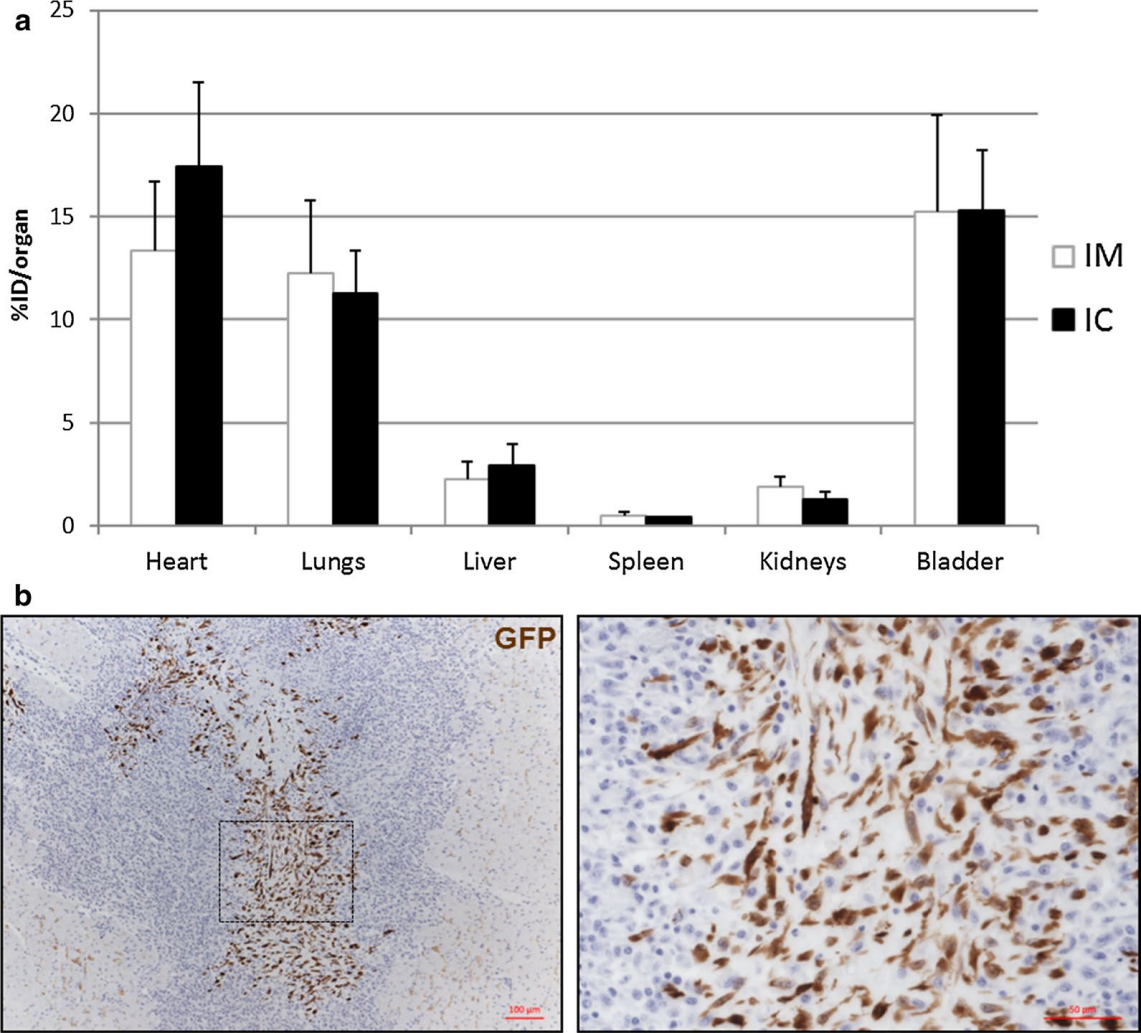
CSC acute retention and engraftment: PET quantitative results (**a**). Data is shown as %ID in every organ, after 4 h of IM (n = 3) or IC (n = 3) administration of ^18^F-FDG labelled cells. Data is represented as mean plus standard deviation. Representative images of engrafted GFP^+^ cells in the heart (**b**). Tissue sections were immunohistochemically stained for detection of GFP^+^-CSC (in *brown*), 3 days post-implant. Positive cells were found in the hearts of all the IM implanted pigs, whereas no cells were found in any of the IC injected pigs. A representative picture (and magnification) shows the presence of engrafted CSC in the myocardium of an IM implanted pig

Quantitative analysis of images is summarized in Fig. 4a and Additional file 3: Table S1. No statistical differences in %ID were found between the IM and IC routes of administration in the different organs. The largest activity attributable to cells was located in the heart and no statistically significant differences in %ID were found between animals that received the cells IM (13.4 ± 3.4%) or IC (17.4 ± 4.1%) (p = 0.27). High values of %ID were also observed in lungs (IM 12.2 ± 3.5%; IC 11.3 ± 2.1%) (p = 0.82). The rest of the activity attributable to free-^18^F-FDG was mainly located in the bladder (IM 15.2 ± 4.7%; IC 15.3 ± 2.9%) (p = 0.83), and to a lesser extent in the kidneys. Radioactivity accumulation in liver and spleen was very low in comparison with other organs.

Taking into account the physiological biodistribution of free ^18^FDG, and assuming that the vast majority of the cells were entrapped in heart and lungs, the percentage of retained cells in both organs was calculated considering only the radioactivity in these organs. By IM administration, 52.2 ± 13.3% of the cells were located in the heart, whereas this percentage was 60.2 ± 9.7% for IC administration (p = 0.45). The percentage of cell retention in lungs was 47.8 ± 13.3 and 39.8 ± 9.7 for IM and IC administration respectively (p = 0.44).

### Cell engraftment

Cell engraftment was analyzed three days post-transplantation. Immunohistochemical analysis of GFP positive cells in the scar and perinfarcted areas revealed the presence in the myocardium of engrafted CSC in all the hearts that were implanted IM (8.9 × 10^3^, 23.4 × 10^3^, 131.6 × 10^3^ cells/mm^2^). On the contrary, no positive cells were found after IC implant in any of the three pigs (Fig. 4b).

## Discussion

The development of methods for non-invasively monitoring stem cell distribution after transplantation in patients with cardiac diseases is a pre-requisite to compare different delivery systems and potentially, to improve our understanding of the underlying mechanisms. In this study, using a last generation PET/CT hybrid scanner, we compared the tissue distribution and retention of allogeneic CSC in a large animal model of chronic ischemia–reperfusion after two different cell delivery routes (NOGA^®^-guided IM and IC). The combination of PET and CT provided excellent images, significantly better than those obtained in previous studies in swine models of MI [16–18], which allowed more robust and reliable quantification of the implanted cells.

In this study, 50 million cells were injected in our chronic model of MI with no adverse effects detected during or after transplantation by either of the two delivery routes. Previous studies performed in pig acute and subacute infarct IC injected with 25 million CSC have also shown safety and also efficacy [6]. In our model, the quantified images showed that cell retention in heart was equivalent but with a clearly different pattern between IC and NOGA^®^-guided IM injection, which reflects the delivery route. Other studies have also compared by means of cell radiolabeling and imaging tracking several delivery methods for stem cells in MI animal models [13–18]. Interestingly, Hou and collaborators directly compared different delivery methods (IC, IM and interstitial retrograde coronary venous delivery) in a swine infarct model [16]. In this case, %ID values detected in heart were comparable to our results when IM injected (about 12%). However, IC delivery and interstitial retrograde coronary venous delivery gave rise to somewhat lower retention values (about 3%). This discrepancy in the IC values between these two preclinical studies might well be due to methodological differences including cell type, time of post-infarction treatment and imaging methods that do not allow a direct comparison of the results.

Beside the heart, we detected a large accumulation of activity in bladder and kidneys (renal medulla), whereas liver and spleen had a very low signal. Kidney and urinary bladder activities reflected the excretion of free ^18^F-FDG. Moreover, not unexpectedly, our results also showed that a large percentage of cells was retained in the lungs, also independently of the route of administration. After an intravenous injection of free ^18^F-FDG, cardiac uptake is variable, but most frequently mild and always homogeneous; moreover, it is well known that the lungs normally exhibit very low ^18^F-FDG uptake [23]. Our images showed a different pattern of activity, so it can be assumed that the signal attributable to the radiolabeled cells is almost completely located in heart and lungs. The signal accumulation in the lungs suggests that a percentage of cells were not retained at the heart, and were later entrapped in the lungs [13, 15, 16, 18]. It has been suggested that this largely right-sided distribution of cells, marked by pulmonary cell trapping far in excess of that by filter organs like liver and spleen, could be caused by an egress of cells into the myocardial venous or lymphatic drainage rather than into the arterial conduits [16]. Finally, the uptake in some mediastinal lymph nodes could be the result of lymphatic drainage from the injection site or the presence of some reactive lymph nodes.

Unlike cell retention, cell engraftment determined by immunohistological detection of GFP^+^-CSC at 3 days post-transplant showed significant differences between IC and IM administration. NOGA^®^-guided IM injection favored cell engraftment in comparison with IC injection as engrafted CSC were detected 3 days post-implantation in all the IM-implanted pigs whereas no cells were found in any of the IC-implanted animals. Although a certain degree of cell engraftment cannot be fully discarded, a clear discrepancy between the PET and histological results was found, which could be explained as a result of faster cell attrition after IC delivery. PET/CT images correspond to the first hours post-implantation, a phase in which cells are distributed and retained in the tissues. However, cells are swiftly and acutely lost from the tissue due to mechanical washout, which impairs their persistence and engraftment into the tissue, and that may depend on the chosen delivery route [13, 24]. In fact, the improved engraftment detected after NOGA^®^-guided IM cell injection is consistent with previous studies where several (stem) cell populations and several delivery routes (intravenous, IM or IC routes) have been comparatively analyzed in MI animal models. In those studies, a greater engraftment was found when using the IM route [3, 11]. Importantly, a greater functional improvement has been observed by others and also by our group when greater cell engraftment is achieved [20, 25–27]. In any case, although the direct correlation between engraftment and therapeutic benefit is expected, this does not necessarily always correlate. In our study, a greater engraftment was detected after IM injection. However, taking into account the similar rate of short-term retained cells and that the IC route allows a broader cells distribution (which could propitiate a short but robust paracrine effect of the implanted CSC [28]), functional long-term analysis will be also needed in order to confirm which cell delivery method might be more efficient as a therapeutic treatment. Therefore, we are performing new functional long-term studies in our animal model with that purpose. All this comparative data can be especially relevant in order to treat those patients, mainly the chronic ones, in which the coronary artery might be blocked and hence, it would not be possible to implant the cells via the IC route.

In this study, allogeneic instead of autologous cells were implanted. Limitations of the autologous approach related with the efficacy of the cells due to the patient´s age and disease as well as time, logistic and economic issues for cell production, makes of the allogeneic approach an interesting option for stem cell therapy. However, in the case of allogeneic treatments, the immunogenic response may limit the time during which the cells may exert their effect. Therefore, the immunogenic profile that allows the cells to remain in the tissue is a key issue that needs to be elucidated in order to reach a beneficial effect. Importantly, we have previously demonstrated the CSC tolerogenic immune behavior in the allogeneic setting [29]. CSC, as well as the mesenchymal stem cells, are characterized by the expression of HLA-class I, but lack of expression of HLA-class II and the co-stimulatory molecules CD40, CD80 or CD86. Previous in vitro studies have also confirmed their low immunogenic profile, suggesting that CSC, although recognized by the immune system, will be initially tolerated [29]. Importantly, a functional benefit of the allogeneic CSC has been also demonstrated by our group in a preclinical model of acute MI in pig [6]. All these data show the immunotolerogenicity of CSC which favors their retention despite their allogeneic origin and therefore, their therapeutic benefit. Interestingly, the immunotolerogenic capacity of our CSC population has been shown also in other CSC populations like the cardiosphere-derived cells. Also with this other particular cardiac cell population, safety and a functional benefit has been shown after the allogeneic implant in rat and pig models of MI [30–32]. In view of these results, two phase I/II clinical trials (CAREMI trial (NCT: 02439398) and ALLSTAR trial [33]) are ongoing in order to determine the safety and efficacy of the allogeneic CSC and cardiosphere-derived cells respectively, as a treatment for patients with acute MI and ischemic left ventricular dysfunction.

Finally, this study has some limitations. On the one hand, although the data clearly show the different patterns of cell biodistribution and retention, it would have been optimal to include a greater number of animals in order to perform a more robust statistical analysis. On the other hand, the use of ^18^F-FDG labeling to track stem cells offers some advantages like the simplicity of the labeling method, its great availability and the potential to track cells with PET/CT even in the clinical setting. However, this procedure presents some disadvantages that cannot be overlooked. First, ^18^F-FDG labeling only allows short-term tracking of the cells due to the half-life of the tracer (T_1/2_ = 109.8 min) and second, ^18^F-FDG can in part be released from labeled cells. Classic methods for cell labeling in the clinical setting include the use of gamma emitting radioisotopes like Indium-111 (T_1/2_ = 2.80 days). However, monitoring only can be performed using single photon emission computed tomography (SPECT/CT), a technique that provides images with significantly lower resolution and sensibility than those obtained by PET/CT. Very recently, some groups have developed different methods of cell labeling with ^89^Zn, a positron emitter radionuclide with a longer half-life (T_1/2_ = 78.4 h). Cell labeling strategies include the transport of ^89^Zr into cells in conjunction with oxine [34] or the direct labeling of cells surface by the creation of labeling entities with different chelators [35]. Those methods allow stable labeling of a wide range of cellular types that can be in vivo tracked by PET/CT imaging for up to 7 days. The development of cell labeling techniques based on the use of other radiotracers like ^52^Mn (T_1/2_ = 5.6 days) is also under study [36]. In that sense, it would be interesting for future studies to use this type of radionuclides that would allow the assessment of longer-term cell engraftment in our animal model.

## Conclusion

In conclusion, we demonstrate that PET/CT images can accurately determine the biodistribution of ^18^F-FDG labeled cells in a large animal. Although acute retention of CSC is similar after either IM or IC implantation this does not translate into a similar level of cell engraftment, since this is greater after NOGA^®^-IM injection. Considering previous preclinical and clinical data that have shown the benefits of IC transplantation of CSC in acute MI in terms of safety and therapeutics [6], further research is needed to explore the comparative analysis of allogenic CSC delivered by NOGA^®^-IM injection for the chronic pathology.

## Additional files

**Additional file 1.** PET IC delivery.

**Additional file 2.** PET IM delivery.

**Additional file 3: Table S1.** PET image quantification results expressed as ID% per organ.

## Abbreviations

CSC: cardiac stem/progenitor cells
FWHM: full width half maximum
GFP: green fluorescent protein
IC: intracoronary
ID: injected dose
IM: intramyocardial
MI: myocardial infarction
PET/CT: hybrid positron emission tomography/computed tomography
SPECT/CT: hybrid single photon emission computed tomography/computed tomography
T_1/2_: half-life
^18^F-FDG: 2-deoxy-2-[^18^F]fluoro-d-glucose
%ID: percentage of injected dose
