# Supplementary material for: Non-invasive in vivo imaging of cardiac stem/progenitor cell biodistribution and retention after intracoronary and intramyocardial delivery in a swine model of chronic ischemia reperfusion injury
Source: J Transl Med. 2017 Mar 13;15:56. doi: 10.1186/s12967-017-1157-0 (PMC5347835; doi:10.1186/s12967-017-1157-0)
Supplement: Supplementary file 3 — Additional file 3: Table S1. PET image quantification results expressed as ID% per organ. [file 12967_2017_1157_MOESM3_ESM.docx]

**Table S1.**

**PET image quantification results expressed as ID% per organ**

| **% ID per organ (mean ± SD )** | | | | | | | |  |
| --- | --- | --- | --- | --- | --- | --- | --- | --- |
|  | | | | | | | |  |
| ID | Heart | Lungs | Liver | Spleen | Lymph nodes | Kidneys | Bladder | |
| IM (n=3) | 13.4 ± 3.4 | 12.2 ±3.5 | 2.3 ± 0.9 | 0.5 ± 0.2 | 1.5 ± 1.1 | 1.9 ± 0.5 | 15.2 ± 4.7 | |
| IC (n=3) | 17.4 ± 4.1 | 11.3 ± 2.1 | 3.0 ± 1 | 0.5 ± 0.1 | 0.1 | 1.3 ± 0.4 | 15.3 ± 2.9 | |
| *p* (IM *vs.* IC) | 0.27 | 0.82 | 0.27 | 1.0 | 0.18 | 0.13 | 0.83 | |
|  | | | | | | | |  |
| **% ID per organ and per animal** | | | | | | | |  |
|  | | | | | | | |  |
| ID | Heart | Lungs | Liver | Spleen | Lymph nodes | Kidneys | Bladder | |
| IM - 1 | 9.5 | 16.3 | 3.0 | 0.7 | 0.7 | 1.7 | 16.5 | |
| IM - 2 | 15.4 | 10.7 | 2.5 | 0.3 | 1.1 | 2.5 | 10.0 | |
| IM - 3 | 15.2 | 9.8 | 1.3 | 0.5 | 2.7 | 1.5 | 19.2 | |
| IC - 1 | 22.0 | 8.9 | 3.8 | 0.4 | 0.0 | 1.6 | 13.0 | |
| IC - 2 | 14.1 | 12.6 | 3.3 | 0.5 | 0.0 | 1.4 | 14.5 | |
| IC - 3 | 16.2 | 12.3 | 1.8 | 0.5 | 0.1 | 0.8 | 18.5 | |

Data are showed as mean ± SD for IM and IC administration of cells. The p values were calculated using Mann-Whitney U test. Additionally, individual data for each experimental animal are presented.
